# Supplementary material for: Overexpression of Toxic Poly(Glycine-Alanine) Aggregates in Primary Neuronal Cultures Induces Time-Dependent Autophagic and Synaptic Alterations but Subtle Activity Impairments
Source: Cells. 2024 Aug 3;13(15):1300. doi: 10.3390/cells13151300 (PMC11311834; doi:10.3390/cells13151300)
Supplement: Supplementary file 1 [file cells-13-01300-s001.zip › cells-3115693-supplementary.pdf]

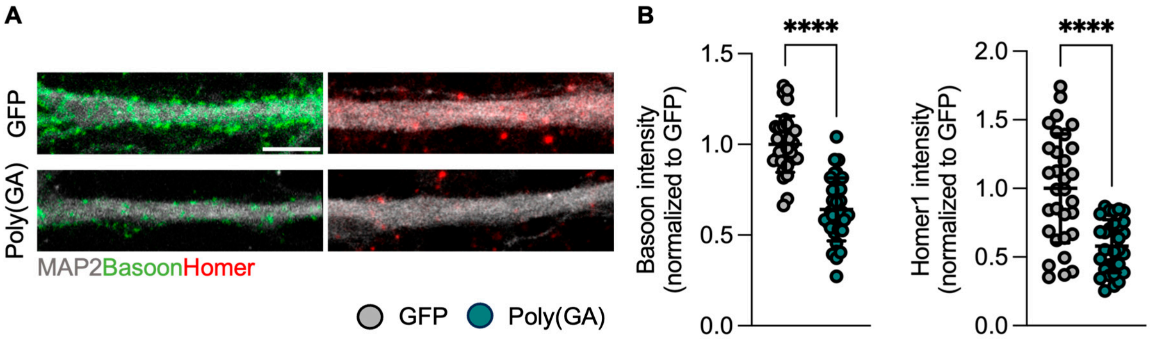

Supplementary Figure S1. Poly(GA)-transduced cells show reduced abundance of synaptic proteins. (A) Representative images of dendrites from polyGA- or GFP-transduced neurons. (B) Quantitative analysis reveals a significantly decreased mean immunofluorescence intensity of Homer (red) and Basoon (green) puncta in neurons accumulating poly(GA)-aggregates (Basoon: PolyGA vs. GFP p<0.0001 ; Homer1: PolyGA vs. GFP p<0.0001). MAP2 is represented in gray. Values are shown as fold change to GFP. Basoon and Homer puncta were analyzed along a dendrite length of 10µm. Experiments were performed in n=3 independent replicates. Data are displayed as mean value ± SD (Welch's t-test; ****p<0.0001). Scale bar represents 5µm.

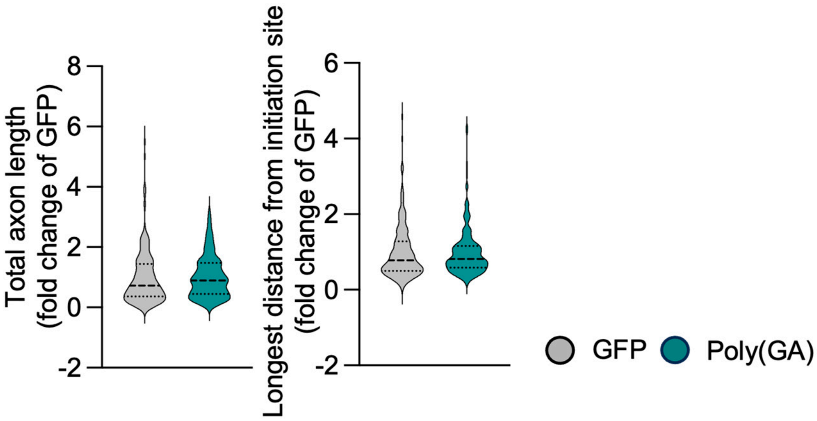

Supplementary Figure S2. Morphological MEA analysis are not affected by poly(GA)-overexpression. Total axon length and longest distance from the initiation site do not show defective signs triggered by toxic poly(GA)-aggregates. Values are shown as fold change to GFP. Experiments were performed in n=3 independent replicates. Data are displayed as mean value ± SD (Welch's t-test; comparisons are not significant).
